# Four-strand hamstring graft is stiffer than a tripled semitendinosus graft in anterior cruciate ligament reconstruction: a cadaveric study

**DOI:** 10.1186/s40634-020-00254-6

**Published:** 2020-05-28

**Authors:** Frans J. A. Hagemans, Karlijn L. J. van Overvest, Jacco A. C. Zijl, Duncan E. Meuffels

**Affiliations:** 1 Department of Orthopaedics and Centre for Orthopaedic Research Alkmaar (CORAL), Northwest clinics, Wilhelminalaan 12, NL – 1815 JD, Alkmaar, The Netherlands; 2 Department of Orthopaedic Surgery, Erasmus MC, University Medical Centre Rotterdam, the Netherlands, Doctor Molewaterplein 40, NL- 3015 GD, Rotterdam, The Netherlands; 3 Department of Orthopaedic Surgery, Antonius Hospital, Nieuwegein Koekoekslaan 1, NL - 3435 CM, Nieuwegein, The Netherlands

**Keywords:** Anterior cruciate ligament reconstruction, Biomechanics: tripled graft, Four-strand graft

## Abstract

**Purpose:**

The aim of this study was to compare the biomechanics of a four-strand hamstring graft with a tripled semitendinosus graft, with and without adjustable extra-cortical button fixation, in a cadaveric model.

**Methods:**

Four groups of 10 cadaveric hamstrings were tested: In group A, a tripled semitendinosus graft fixated with two adjustable extra-cortical buttons; in Group B, a four-strand semitendinosus and gracilis graft fixated with an adjustable extra-cortical button and a clamp; in group C, a tripled semitendinosus graft fixated to a steel hook and a clamp; in group D, a four-strand semitendinosus and gracilis graft fixated to a steel hook and a clamp. Each group was submitted to a cyclic loading test (1000 cycles between 50 and 250 Newton at a frequency of 0.5 hertz) and a load-to-failure test. Primary outcomes were ultimate failure load and stiffness. Secondary outcomes were graft elongation and graft diameter.

**Results:**

There was no difference in ultimate failure load among groups. Group B achieved a median stiffness of 171 N/mm (interquartile range [IQR] 139–204) which was significantly higher than Group A (median 103 N/mm (74–119), *p* < 0.01). Group B showed more cyclic elongation (4.1 mm (3.4–5.7)) compared to group D (2.3 mm (1.9–3.0)), and also lower stiffness was noted (171 N/mm (139–204) vs 265 N/mm (227–305)). There was no difference in graft diameter among groups.

**Conclusions:**

The results of this study indicate that higher stiffness can be achieved using four-strand hamstring tendon grafts compared to tripled semitendinosus grafts when using femoral extra-cortical buttons, despite comparable graft diameters. Thereby, the use of adjustable extra-cortical fixation devices may result in more cyclic elongation and lower stiffness of the graft.

## Introduction

A challenge in anterior cruciate ligament (ACL) reconstruction is to maximize functional outcome and to minimize donor site morbidity. In the history of ACL reconstruction, many graft options have been described, but graft choice remains a contested topic [13]. The past decade has seen a growing trend towards hamstring tendon graft techniques [11]. In Scandinavia, 59% to 90% of all ACL reconstructions are being done by using hamstring tendon grafts [2, 5]. Despite a somewhat higher revision rate in long-term follow-up, hamstring tendon grafts have shown excellent outcomes and are comparable to those of patellar tendon grafts [5, 18].

Still, there are various possibilities for hamstring graft preparation and graft fixation. One of the advancements in graft fixation techniques is the use of adjustable extra-cortical fixation devices. These devices allow the use of bone sockets instead of full tunnels, which reduces bone loss in the tibial tunnel and also reduces the required length of the graft. Because of this advancement, there has been an increasing interest in harvesting only the semitendinosus tendon (ST) and leaving the gracilis tendon intact. Several studies suggested that this approach may reduce donor site morbidity and nerve injury and that it may also result in a better postoperative stability and higher post-operative hamstring muscle strength [6, 14, 17, 19]. However, the clinical relevance of these potential benefits is still unclear.

The tripled ST grafts are used in clinical practice alongside the more widely used four-strand hamstring (doubled semitendinosus and gracilis (STG)) grafts. However, there is a paucity of evidence on the biomechanical properties of tripled ST grafts compared to those of doubled STG grafts.

Therefore, the aim of this study was to evaluate the differences in failure load and stiffness between the all-inside-technique using a tripled semitendinosus graft and a four-strand hamstring tendon (doubled semitendinosus and gracilis) graft technique in a biomechanical setting. Secondary outcome parameters were to determine graft elongation and failure mode of both the tripled versus four-strand construct with an adjustable extra-cortical fixation versus a fixed hook construct. Our hypothesis was that a tripled semitendinosus graft results in a lower failure load and a lower stiffness of the construct compared to a doubled semitendinosus and gracilis tendon graft.

The study was approved by the ethical review board and all patients gave their informed consent.

## Methods

This biomechanical study was executed at the Erasmus MC, University Medical Centre Rotterdam department of Orthopaedic Surgery between January 2017 and January 2018. Forty fresh frozen cadaveric hamstring tendons were harvested by an orthopaedic resident (FH) and orthopaedic surgeon (DM). Each tendon was obtained via the National body donation program, and as such, the use of these tendons was approved by the ethical review board. The tendons were selected on adequate length (≥21 cm) and good quality on visual inspection. Each tendon was sharply trimmed parallel to the fibre orientations and cleaned of all adherent muscle fibres. Afterwards, the tendons were stored in airtight bags in a freezer at − 20 °C until the day of testing. Before testing, tendons were thawed in gauzes with saline and an independent researcher randomly assigned the tendons into four test groups. After graft preparation, the diameter and length of the grafts were measured. Tests were performed at room temperature (18 °C), and all tendons were kept moist with physiological saline during preparation and testing.

The all-inside technique using a tripled semitendinosus tendon and the four-strand hamstring technique were simulated and tested with extra-cortical buttons in an isolated construction. For each technique that was tested, one group was added in which the adjustable extra-cortical fixation device was replaced by a steel hook at the femoral side and by a clamp at the tibial side. The steel hook should not allow elongation and the clamp should not allow for tendon slippage. In this manner, we could determine to what extent the extra-cortical fixation device influences the biomechanical properties. Thus, 4 test groups were compared with each other.

For adjustable extra-cortical fixation, TightRopes RT (Arthrex, Naples, Florida, USA) were used. To simulate the femoral cortex, a custom device was designed of stainless steel. In this device a tunnel was created with a diameter corresponding to the manufacturer’s recommendations (4.0 mm). The tibial side was secured with a clamp with small dents, thus assuring a firm grip on the tendon.

### Samples preparation

#### Group A

**Fig. 1:**
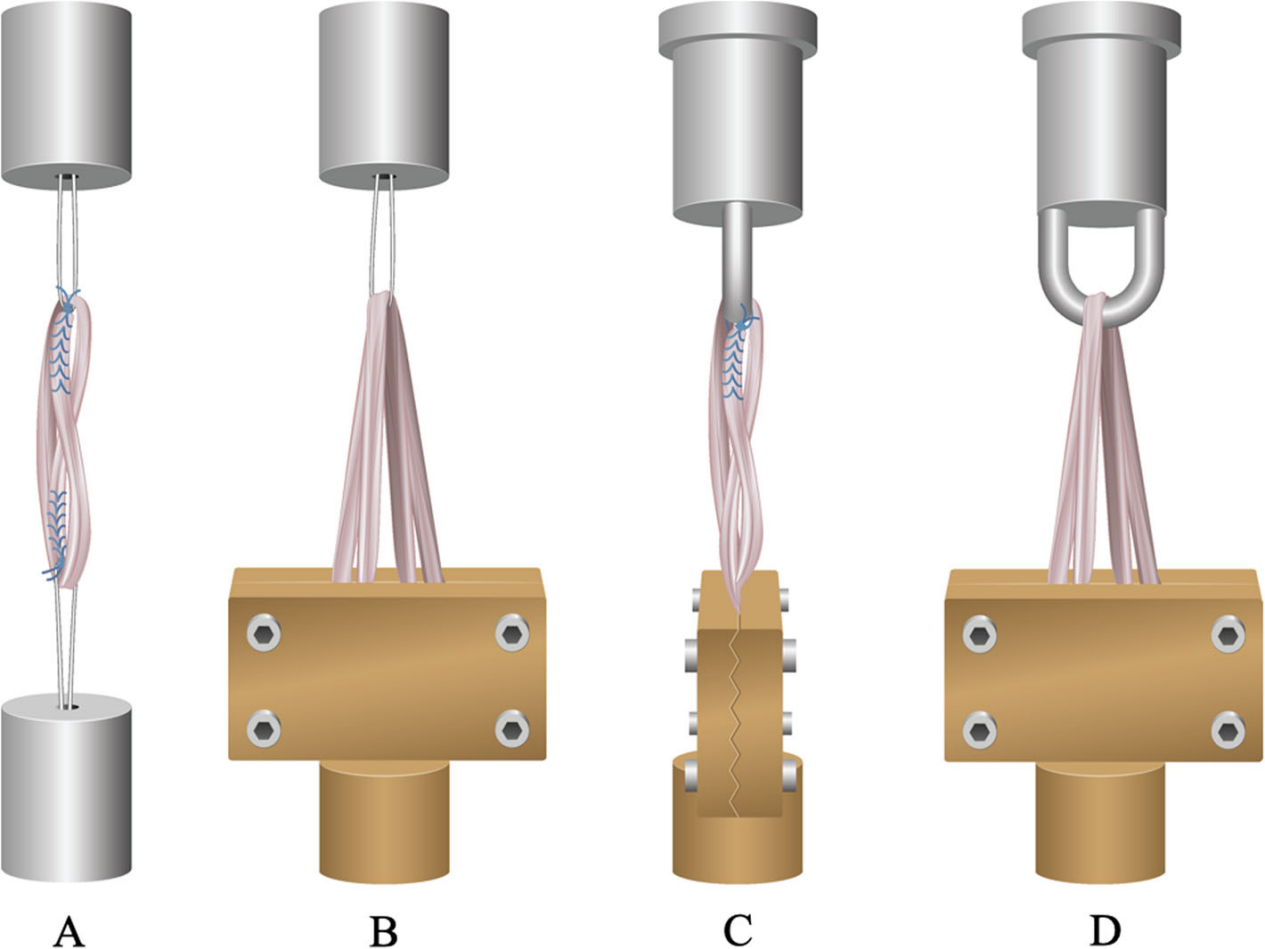
Schematic view of all test groups. Group A (**a**); a tripled semitendinosus tendon (ST) graft with double TightRope fixation; Group B (**b**); a four-strand semitendinosus gracilis (STG) graft with clamp and TightRope fixation; Group C (**c**); a tripled ST graft with clamp and steel hook fixation; Group D (**d**), a four-strand STG graft with clamp and steel hook fixation

A tripled ST graft technique was simulated in the first group, using TightRopes (TR) for fixation. First, the grafts were whipstitched six times up and down through both ends with insoluble sutures (FiberWire, Arthrex, Naples, Florida, USA). Next, the semitendinosus graft was folded over the extra-cortical button loop at the proximal (femoral) side, and then it was folded over the extra-cortical button loop at the distal (tibial) side. Finally, the insoluble sutures of both ends of the graft were knotted via seven alternating knots to the loop of the adjustable extra-cortical button at the tibial side and the femoral side. The two TightRopes were attached to the metal fixation devices [Fig. 1a]. The length of the loop of the TightRopes was kept constant (2 cm) and the loop was secured through knotting the white tensioning strands upon the extra-cortical button. Graft length was 6 to 7 cm.

#### Group B

The second technique was the commonly used four-strand hamstring graft technique, fixated with a TightRope at the proximal (femoral) side and fixated with a clamp at the distal (tibial) side. The STG tendons were symmetrically folded over the extra-cortical button loop at the proximal side, which was attached to the extra-cortical device. Then, the four strands were fixated into the clamp at the distal side. During fixation of the graft into the clamp, all the ends of the strands were whipstitched with sutures and then pulled through the clamp. In this way, it was attempted to divide all strengths equally over each strand [Fig. 1b]. Graft length was 6 to 7 cm.

To determine the biomechanical influences of the TightRope, two groups were added in which we substituted the TightRope with a firm stainless steel hook at the proximal side.

#### Group C

A tripled semitendinosus graft was combined with the steel hook. The ST graft was folded over the steel hook at the femoral side, then a loop was made at the tibial side, and the proximal end of the third strand was knotted to the steel hook with a whipstitched FiberWire. The distal end of the graft was fixated into the clamp at the tibial side [Fig. 1c].

#### Group D

A four-strand STG graft was folded symmetrically through the steel hook at the proximal side. Then it was fixated into the clamp at the distal side. Again, it was attempted to divide all strengths equally by pulling at the sutures, which were stitched through the ends of the graft [Fig. 1d].

### Testing

Tests were performed with a tensile testing machine (Testometric 250–2.5AX; Rochdale, England) that could apply a maximum pull-out force of 1000 Newton (N). The grafts were attached to the machine in such a way that the force on the grafts would have been in line with the long axis of a tunnel in clinical practice. In this way, worst-case testing was executed on the ACL reconstructions. All strands of the grafts were tied together in het center of the graft to create circumferential pressure and all grafts were pre-tensioned by hand before they were attached to the testing machine. The testing protocol of Aga et al. was used for cyclic testing and pull-to-failure loading [1]. First, the grafts were preloaded in tension from 10 to 50 Newton at 0.1 Hz for 10 cycles, and then they were loaded between 50 and 250 Newton for 1000 cycles at a frequency of 0.5 hertz. This simulates the reported forces in the ACL during passive extension while walking and during the early rehabilitation protocol of flexion-extension loading on the reconstructed graft [8]. After the cyclic loading protocol, grafts were further displaced at 50 mm/min until failure in order to simulate a sudden overload event of the knee [7].

### Outcomes

The primary outcome was failure load (in Newton) and stiffness (Newton per millimetre). Failure load was measured at the maximum force before failure of the graft. Stiffness was calculated at the steepest point in the force-displacement curve before the maximum failure load. Secondary outcomes included graft elongation (in millimetre), mode of failure and graft diameter (in millimetre). Mode of failure was observed visually and recorded during and after testing. Failure was classified as tendon failure, failure of the adjustable extra-cortical fixation device, suture failure or clamp loosening. If the graft did not fail, this was also noted. Graft elongation was calculated as the difference between graft length after preloading and after 1000 cycles of our testing protocol. Graft diameter was measured with a graft-sizing block (4.5–12 mm holes in 0.5 mm increments - AR-1886, Arthrex).

### Statistical analysis

Sample size calculation was performed and a minimum of 10 per group was sufficient to detect a 20% difference (α = 0.05%) in maximum failure load assuming an SD of 43 with mean maximum failure load of 859 N [10]. Normality of the distribution was assessed by the Shapiro-Wilk test. For failure load, elongation and stiffness (data non-normally distributed), a Kruskal Wallis test was used to test for differences among all test groups and afterwards Mann-Whitney U tests were performed to reveal which groups differed from each other and results were presented as median with interquartile range (IQR). For graft diameter (normally distributed data) one-way ANOVA was used and results were presented in mean and standard deviation (SD).

Comparing the two grafts used in the clinical situation, group A and B were compared. Group C and D were compared to detect differences between a tripled ST graft and a four-strand hamstring graft without the influence of the adjustable fixation device. Group B and D were compared to observe the influence of the adjustable cortical button device.

Statistical analysis was performed using SPSS statistics version 24 (IBM, Chicago, Illinois, USA). The significance level was set at *P* < 0.05.

## Results

### Failure load

**Fig. 2:**
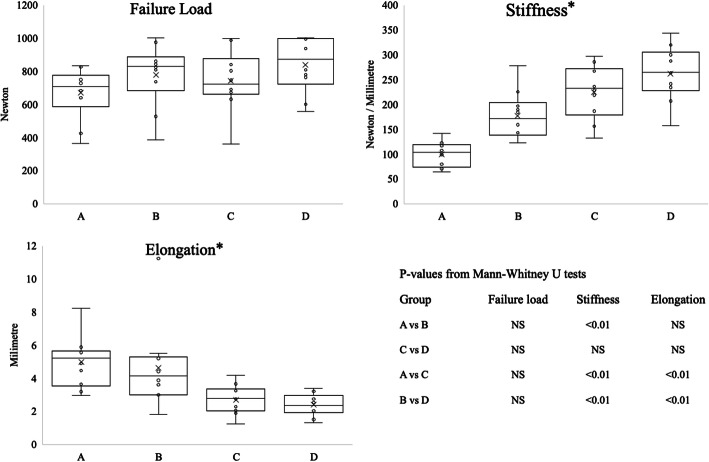
Per group a boxplot is shown which presents the median, interquartile range, maximum and minimum and *P*-values from the Mann-Whitney U tests. The significance level was set at *p* < 0.05. * Significant difference according to Kruskal Wallis test

The highest ultimate failure load was measured in the four-strand group D and group B, followed by the tripled strands in group C and group A, this however was not statistically significant (Table 1). Also, there were no significant differences in failure load between the groups individually [Fig. 2].

**Table 1:** Maximum Failure load, Stiffness, Elongation and Graft Diameter

Groups	Failure load, N	Stiffness, N/mm	Elongation, mm	Graft Diameter, mm^b^
A	708 (587–774)	103 (74–119)	5.2 (3.4–5.7)	8.6 ± 1.1
B	828 (684–888)	171 (139–204)	4.1 (3.0–5.3)	8.6 ± 0.8
C	721 (661–877)	232 (178–272)	2.8 (2.0–3.3)	8.4 ± 0.6
D	873 (723–996)	265 (227–305)	2.3 (1.9–3.0)	8.1 ± 0.4
*P*-value	NS^a^	**< 0.01**^a^	**< 0.01**^a^	NS^b^

Data are presented in median and interquartile range for Failure load, Stiffness and Elongation

^a^Group comparison was performed with a Kruskal Wallis test

^b^Graft diameters are presented in mean and SD and were analyzed with One-way ANOVA

The significance level was set at *P* <  0.05

### Stiffness

Stiffness was highest in the four-strand group D, followed by group C (tripled graft), group B and group A (Table 1). Stiffness in group B (median 171 N/mm (139–204)) was significantly higher than in group A (median 103 N/mm (74–119), *p* < 0.01). Furthermore, our groups using a steel hook for femoral fixation, group C and D, achieved higher stiffness compared to group A and B respectively [Fig. 2].

### Elongation

No grafts failed during the cyclic testing from 50 N to 250 N. The lowest amount of total elongation after 1000 cycles was observed in group D with a median elongation of 2.3 mm (1.9–3.0), followed by group C with 2.8 mm (2.0–3.3), group B 4.1 mm (3.0–5.3) and group A with 5.2 mm (3.4–5.7) (Table 1). Group B showed more elongation than group D, this difference was also noted between group A and C [Fig. 2].

### Graft diameter and mode of failure

**Fig. 3:**
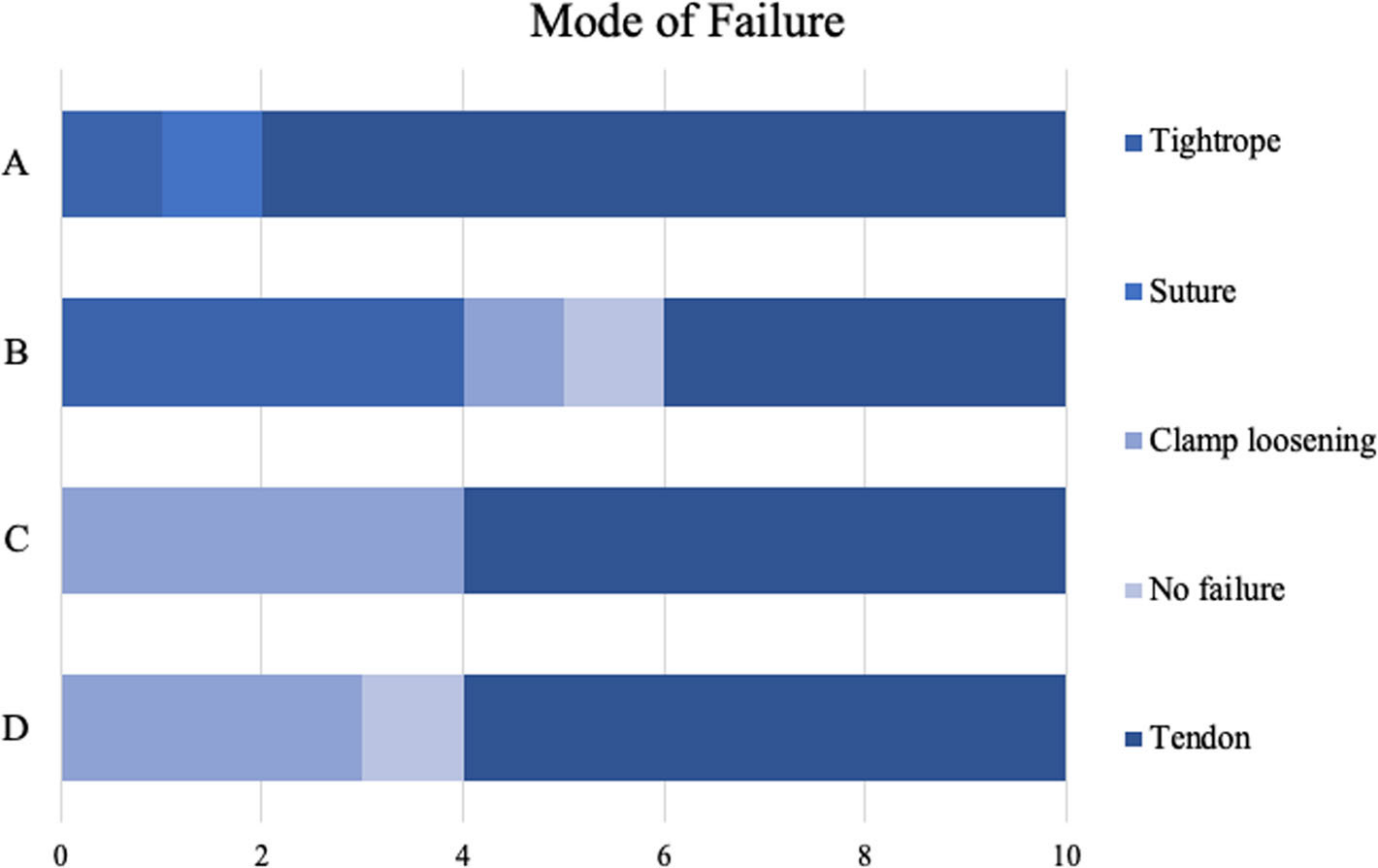
Graphic view of mode of failure per group. In group A no clamp was used. For group C and D only clamp loosening and tendon failure is applicable

There was no difference in graft diameter between the tripled semitendinosus grafts and the four-strand hamstrings grafts (Table 1). In group A, the majority of tendons, eight out of 10, failed due to tendon rupture [Fig. 2]. The tendons ruptured at the proximal end at the knot of the FibreWire and on the loop of the TightRope at the femoral side. In group B, four out of 10 TightRopes failed [Fig. 3]. Of these failures, the average failure load was 864 N.

## Discussion

The main finding of this study was that four-strand HT grafts fixated with an adjustable extra-cortical button device at femoral side result in higher stiffness compared to tripled ST grafts with use of dual adjustable extra-cortical fixation devices. Furthermore, there was no difference in maximum failure load or graft diameter.

Despite that our four-strand groups revealed a trend towards failure loads, these differences were not significant. However, all the observed failure loads were far above the forces applied during rehabilitation (250 N- 303 N) as reported by Rupp and Shelburne [12, 15]. The study of Geethan et al., who compared four-strand grafts with tripled grafts similar to ours, found higher failure loads in the four-strand graft group [4]. The only difference was that they used only one button fixation instead of dual button fixation for the tripled graft. An explanation for higher failure loads in four-strand grafts might be that the forces applied on the graft are more equally divided. This view is supported by the study of Snow et al., who observed significantly more cyclic elongation in the third limb of a tripled tendon in comparison with a four-strand (doubled tendon) graft and thus might affect failure load [16].

The most important clinically relevant finding of this study was that a higher stiffness was found in group B compared to group A. This is in line with the study of Mayr et al. who found that a four-strand graft with a femoral adjustable extra-cortical button and a tibial interference screw had a higher mean pull-out stiffness than a graft with dual cortical button fixation [9]. However, the difference in stiffness between groups A and B did not result in differences in elongation during cyclic loading. At the same time, stiffness measured in the TightRope groups (A and B) were lower compared to the groups using the steel hook (C and D). This reflects to what extent the TightRope affects the stiffness of the construct, despite the closed loop construction. This might indicate that using two TightRopes will result in a lower stiffness than the use of one TightRope in clinical practice, which is also corroborated by the study of Mayr et al. [9].

Since stiffness is correlated with elongation, we observed greater elongation in the Tightrope groups than in the groups using a steel hook. These results match the ideas of Barrow et al., who found clinically significant increased loop lengthening for adjustable fixation devices during their cyclic loading tests [3]. This lengthening may be partly explained by suture slippage into the adjustable-length loop and this might influence graft- lengthening during the acute postoperative period. Also, the study of Petre et al. found more cyclic displacement in adjustable-length loop devices compared to fixed loop devices [10]. Therefore, it is not surprising that a significant difference was found of approximately 2 mm more elongation in the Tightrope groups compared to the grafts with a rigid steel hook. However, this must be considered when using adjustable loop devices for both femoral and tibial fixation in the clinical situation.

The findings in our study may be limited by the in vitro biomechanical set up, that we chose for our tests. We have chosen for clamp fixation instead of an interference screw. In group B, only 1 clamp loosening was observed. In group C and D, clamp loosening was observed 7 times, at relatively high failure loads (mean of 791 N) compared to the mean failure load reported by Mayr et al. (694 N ± 119 N). In eight out of 10 cases of their screw fixation group, these authors observed graft slippages from the tibial tunnel [9]. This suggests that the grip of our clamp was stronger than the grip of a potential interference screw fixation. Thereby, the Testometric was limited to a pullout-force of 1000 N that was reached by two four-strand grafts without failure of the construct. For these groups we have underestimated the mean failure load.

The forces that were applied on the grafts were in an axial line, which is not comparable with the in vivo situation where the graft is placed into oblique bone sockets and the forces on the graft are not in an absolute axial direction. Nevertheless, we believe that the forces we applied correspond with the worst-case test scenario. Therefore, we think that in the clinical situation the elongation might be less than in our study.

Another source of uncertainty is the abnormal distribution of our data. This is partly due to the small sample size and partly due to a few outliers. Our results therefore need to be interpreted with caution. Although our study design aimed to minimize confounding factors, we observed a larger width in our data than we expected. If we extrapolate this to the clinical situation, where biomechanical properties are subject to more influencing factors some unknown at the time of surgery, there may be more or less variation in fixation strengths, in distribution of forces on the strands and in stiffness of the construct and outliers could be interpreted as comparable to the clinical setting.

## Conclusions

In this biomechanical study, a four-strand semitendinosus gracilis graft is a stiffer construct compared to a tripled semitendinosus when using adjustable extra-cortical fixation devices, and might therefore be the preferred graft technique. The use of adjustable extra-cortical fixation devices negatively affects stiffness and cyclic elongation of the graft.

## Data Availability

Not applicable.

## Abbreviations

ACL: Anterior cruciate ligament
ST: Semitendinosus tendon
STG: Doubled semitendinosus and gracilis
TR: TightRope
